# Supplementary material for: Sound-seeking before and after hearing loss in mice
Source: bioRxiv. 2024 Jan 9:2024.01.08.574475. Preprint. [Version 1] doi: 10.1101/2024.01.08.574475 (PMC10802496; doi:10.1101/2024.01.08.574475)
Supplement: Supplement 1 [file NIHPP2024.01.08.574475v1-supplement-1.pdf]

bioRxiv preprint doi: https://doi.org/10.1101/2024.01.08.574475; this version posted January 9, 2024. The copyright holder for this preprint (which was not certified by peer review) is the author/funder, who has granted bioRxiv a license to display the preprint in perpetuity. It is made available under aCC-BY 4.0 International license.

SUPPLEMENTAL FIGURES

Supplemental Table 1. Mice used in this study.

Cohort	Group	Strain	Regulation
1	1A	CBA/CaJ;<br>C57BL6/J:CBA/J	deprivation
	1B	CBA/CaJ;<br>C57BL6/J:CBA/J	deprivation
	1C	CBA/CaJ	deprivation
2	2A	C57BL6/J:CBA/CaJ;<br>C57BL6/J:CBA/J	deprivation
	2B	CBA/CaJ	deprivation
3	3A	C57BL6/J:CBA/J	unpalatability
4	4A	C57BL6/J:CBA/CaJ	unpalatability
	4B	C57BL6/J:CBA/CaJ	unpalatability
	4C	C57BL6/J:CBA/CaJ	unpalatability
	4D	C57BL6/J:CBA/CaJ	unpalatability
5	5A	C57BL6/J:CBA/CaJ	unpalatability
	5B	C57BL6/J:CBA/CaJ	unpalatability

This table provides additional information about the mice used in this study, with cohort number and group names matching Table 1 in the main text. The column “Strain” indicates the strain of mouse used in each cohort, with a colon indicating an F1 hybrid of two strains. Groups 1A, 1B, and 2A comprised mice of different strains or hybrids, which are separated with a semicolon and a linebreak in the table. Mice were either purchased directly from Jackson Laboratories or bred in our animal facility. All breeder mice were purchased from Jackson Laboratories, with stock numbers 000664 (C57BL6/J), 000654 (CBA/CaJ), and 000656 (CBA/J).

The column ”Regulation” indicates the water regulation paradigm used. “Deprivation” means that no water was available in the home cage. “Unpalatability” means that mice had ad libitum access in the home cage to water containing 1.5-3% citric acid, which lends a slightly sour flavor. In all cases, rewards during the behavioral task were fresh water from a water purification system (Millipore). Mice were monitored daily for health while on any form of water regulation.

The arena geometry was varied slightly throughout Cohorts 1, 2, and 3. For instance we tried different lengths and heights of the dividers between chambers, and we switched from mounting the speakers on the inside of the wall to a recessed holder instead. Additionally we tried longer inter-trial intervals up to 5 s, switched from one type of speaker (Multicomp Pro MCPCT-G5100-4139) to another (Almencla Ultrasonic Tweeter 2-inch Waterproof Piezo Horn), and refined our speaker calibration procedures. Cohorts 4 and 5 were trained with a consistent arena geometry, finalized speaker calibration, and an inter-trial interval of 1s.

bioRxiv preprint doi: https://doi.org/10.1101/2024.01.08.574475; this version posted January 9, 2024. The copyright holder for this preprint (which was not certified by peer review) is the author/funder, who has granted bioRxiv a license to display the preprint in perpetuity. It is made available under aCC-BY 4.0 International license.

Supplemental Figure 1. Learning across sex and cohort

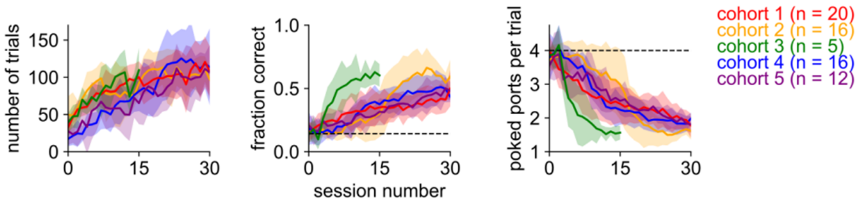

Learning was variable between cohorts of mice but all cohorts reached a roughly similar level of final performance.

bioRxiv preprint doi: https://doi.org/10.1101/2024.01.08.574475; this version posted January 9, 2024. The copyright holder for this preprint (which was not certified by peer review) is the author/funder, who has granted bioRxiv a license to display the preprint in perpetuity. It is made available under aCC-BY 4.0 International license.

Supplemental Figure 2. Acoustic brainstem response (ABR) after hearing loss

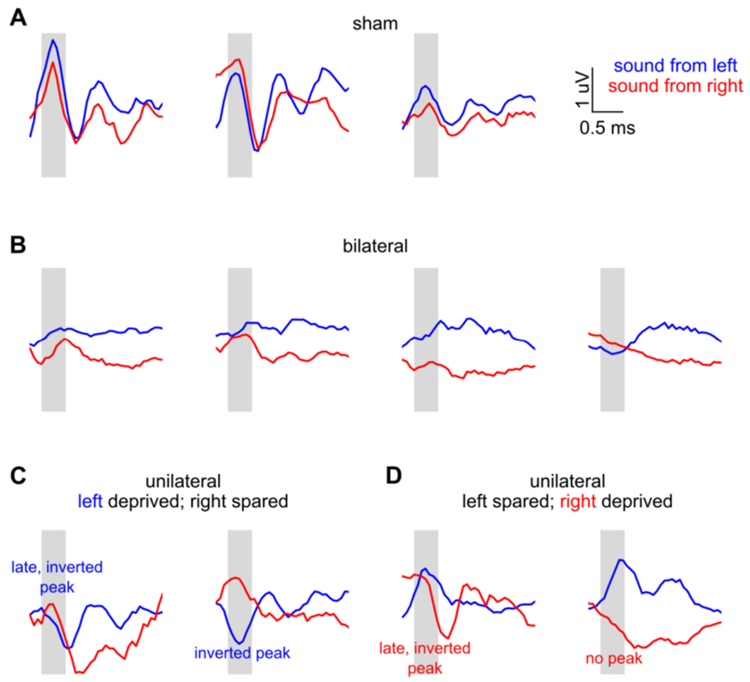

A. ABR following sham hearing loss surgery (control) in response to a click from left (blue) or right (red). This is a differential voltage measured at the left and right ear, averaged over about 1000 clicks. Gray shaded box indicates 2.0 - 2.3 ms after the click, the expected time of the first peak ("Wave 1"). The ABR signal has been inverted for sounds from the left to make Wave 1 positive. All three mice show strong, positive peaks within the shaded box for sounds from both sides.

B. ABR following bilateral hearing loss. Wave 1 is attenuated, delayed, or abolished.

C. ABR following unilateral (left) hearing loss. Wave 1 is delayed or inverted (negative) for sounds from the left (blue). Because of the differential recording procedure, the inversion likely represents a positive response at the other (spared) ear.

D. ABR following unilateral (right) hearing loss. Wave 1 is delayed or inverted (negative) for sounds from the right (red).
